# Preoperative chemotherapy and radiotherapy concomitant to cetuximab in resectable stage IIIB NSCLC: a multicentre phase 2 trial (SAKK 16/08)

**DOI:** 10.1038/s41416-019-0447-0

**Published:** 2019-04-16

**Authors:** Alessandra Curioni-Fontecedro, Jean Yannis Perentes, Hans Gelpke, Alexandros Xyrafas, Hasna Bouchaab, Nicolas Mach, Oscar Matzinger, Nina Stojcheva, Martin Frueh, Walter Weder, Richard Cathomas, Piera Gargiulo, Lukas Bubendorf, Miklos Pless, Daniel Betticher, Solange Peters, for the Swiss Group of Clinical Cancer Research (SAKK)

**Affiliations:** 1 University Hospital of Zurich, Zurich, Switzerland; 2 University Hospitals of Vaud, Lausanne, Switzerland; 3 Cantonal Hospital of Winterthur, Winterthur, Switzerland; 4 SAKK Coordinating Center, Bern, Switzerland; 5 University Hospitals of Geneva, Geneva, Switzerland; 6 Hospital Riviera-Chablais of Vaud-Valais, Vevey, Switzerland; 7 Cantonal Hospital of St. Gallen, St. Gallen and University of Bern, Bern, Switzerland; 8 Cantonal Hospital of Graubünden, Chur, Switzerland; 9 University Hospital of Basel, Basel, Switzerland; 10 Cantonal Hospital of Fribourg, Fribourg, Switzerland

**Keywords:** Surgical oncology, Non-small-cell lung cancer

## Abstract

**Background:**

Neoadjuvant chemotherapy (CT) followed by radiotherapy (RT) and surgery showed a median survival of 28.7 months in resectable stage IIIB non-small-cell lung cancer (NSCLC) patients (pts). Here, we evaluate the impact of concomitant cetuximab to the same neoadjuvant chemo-radiotherapy (CRT) in selected patients (pts) with NSCLC, stage IIIB.

**Methods:**

Resectable stage IIIB NSCLC received three cycles of CT (cisplatin 100 mg/m^2^ and docetaxel 85 mg/m^2^ d1, q3w) followed by RT (44 Gy in 22 fractions) with concomitant cetuximab (250 mg/m^2^, q1w) and subsequent surgery. The primary endpoint was 1-year progression-free survival (PFS).

**Results:**

Sixty-nine pts were included in the trial. Fifty-seven (83%) pts underwent surgery, with complete resection (R0) in 42 (74%) and postoperative 30 day mortality of 3.5%. Responses were: 57% after CT-cetuximab and 64% after CRT-cetuximab. One-year PFS was 50%. Median PFS was 12.0 months (95% CI: 9.0–15.6), median OS was 21.3 months, with a 2- and 3-yr survival of 41% and 30%, respectively.

**Conclusions:**

This is one of the largest prospective phase 2 trial to investigate the role of induction CRT and surgery in resectable stage IIIB disease, and the first adding cetuximab to the neoadjuvant strategy. This trial treatment is feasible with promising response and OS rates, supporting an aggressive approach in selected pts.

## Background

Management of locally advanced NSCLC is still challenging, and the role of surgery is mainly defined by regional preferences and local standards. While definition of stage IIIA/B has been slightly modified through the recent 6th to 8th TMN classification, stage IIIB/C as defined by T4 or N3 disease is characterised by a dismal 5-year overall survival (OS) ranging between 10 and 25%, possibly slightly higher in patients with resectable disease.^1,2^ Previously, several phase 2 trials have evaluated multimodal surgical treatment strategy, including induction chemo-radiotherapy (CRT) followed by surgery for patients with stage IIIB disease. Retrospective subgroup analyses of these trials, particularly of Southwest Oncology Group (SWOG) 8805, suggest that patients with operable stage IIIB NSCLC might present outcomes similar to those with stage IIIA disease.^3^ The Swiss Group for Clinical Cancer Research (SAKK) demonstrated previously that, in well-selected patients, the use of neoadjuvant chemotherapy (CT) of docetaxel/cisplatin followed by neoadjuvant accelerated radiotherapy (RT) of 44 Gy was feasible.^4^ Promising results of this SAKK 16/01 trial and surgical series^5,6^ are the rationale for the current quadrimodal concept, introducing cetuximab to neoadjuvant CRT. Cetuximab was a promising targeted agent in NSCLC, at the time of designing this clinical trial^7^ and in general in combination with RT.^8–10^ No unexpected toxicities were reported by combining platinum-based CT and cetuximab, or by adding cetuximab to definitive CRT in NSCLC.^11,12^ This is the first study to assess the feasibility and activity of adding cetuximab in the induction part of a trimodality treatment for stage IIIB NSCLC. The main objective of this trial was to evaluate efficacy and safety in patients with operable stage IIIB NSCLC.

## Methods

### Patients

Patients were enroled from 11 participating sites, with experience in trimodality-approach, into this open-label, multicentre, prospective phase 2 SAKK trial. Patients with resectable stage IIIB (T4N0–3M0 or T1–4N3M0), as evaluated by a multidisciplinary tumour-board at diagnosis and according to 6th TNM classification, were considered eligible. Stages IIIB with malignant pleural or pericardial effusion, invasion of the aorta, oesophagus, myocardium, supraclavicular, scalene N3 nodes, or with satellite lesions in the same lobe as only T4 descriptor were excluded. Baseline assessment was performed by brain magnetic resonance imaging, whole body PET-CT, contrast enhanced CT scan of thorax and upper abdomen, pulmonary function test and electrocardiogram. Lymph-node staging was performed by mediastinoscopy or EBUS in cases of N-positive disease on PET-CT (SUV above mediastinum background SUV) or CT (size of >10 mm in the smallest diameter), within 42 days from registration. In case of lymph nodes not accessible by mediastinoscopy (ATS position 5 or 6), fine needle aspiration biopsy by EBUS, TBNA or VATS was required.^13,14^ In case of N-negative disease on PET-CT and CT, mediastinoscopy was only mandatory in case of suspicion of T4 tumour invading the trachea. Patients had to be medically fit to undergo surgery and to present sufficient pulmonary reserve to allow the required surgery, according to ESTS guidelines.^15–17^ Other eligibility criteria included: age 18–75 years; WHO performance status 0 or 1; adequate bone marrow, hepatic and renal function, as well as heart function within 42 days from registration.^18^ The study was done in accordance with the principles of the Declaration of Helsinki. The protocol was approved by the ethics committee of each participating site. Written informed consent was obtained from all patients.

### Procedures

**Fig. 1:**
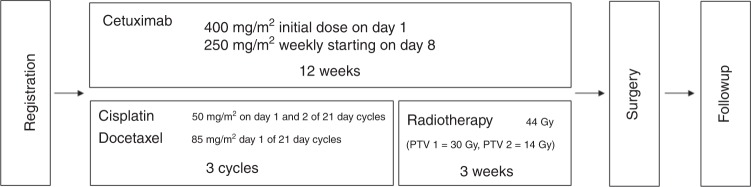
Study design

CT consisted of three cycles of intravenous cisplatin (day 1 and 2, 50 mg/m²) and docetaxel (85 mg/m² day 1) given every 3 weeks. The administration of prophylactic granulocyte-colony stimulating factor was compulsory. Dose reductions were not allowed for cisplatin. Switch to carboplatin (target area under the curve 6) was allowed if patients developed renal function impairment (creatinine clearance lower than 50 mL/min), hearing loss worse ≥grade 2, or peripheral neuropathy ≥grade 3. Cetuximab was given weekly at an initial loading dose of 400 mg/m² and then at 250 mg/m² during the entire course of CT and RT. After cetuximab-CT and before radiotherapy, positron emission tomography–computed tomography (PET-CT), contrast enhanced computed-tomography scan of thorax and upper abdomen, pulmonary function testing and diffusion capacity were repeated. RT was planned in all patients without progressive disease (PD) after cetuximab-CT and started 3 weeks after the last CT administration. The regimen consisted of a total dose of 44 Gy in 22 fractions of 2 Gy (PTV 1 = 30 Gy, PTV 2 = 14 Gy). The overall treatment duration was 19 days. Dose prescription and recording had to comply with the recommendations of the ICRU 50/62 quality control. Before surgery, contrast enhanced CT scan of thorax and upper abdomen and pulmonary function tests were obtained and each patient was evaluated by a local multidisciplinary tumour-board for surgical resection. Surgery was to be planned within 21–28 days after completion of RT (study design is represented in Fig. 1). The recommendation was to avoid pneumonectomy whenever possible, especially in situations where a complete R0 resection could be obtained by a parenchyma-saving procedure. Surgery included tumour resection and systematic lymph-node dissection. Patients whose tumours had progressed at either post-baseline assessment were withdrawn from the study treatment but further followed for toxicity and survival. Patients attended follow-up visits 1 month after surgery, then every 3 months for 2 years, every 6 months for 3 years, until 5 years after surgery or treatment termination, unless clinically indicated otherwise. During visits patients were assessed for toxicity and chest CT every 3 months for the first 2 years, afterwards every 6 months until 5 years. WHO criteria were used to assess tumour response; assessments were done locally by the trial investigators. Adverse events were graded according to the 1994 revised version 3.0 of the National Cancer Institute of Health CTCAE guidelines. Moreover, the first 25 patients were strictly evaluated regarding safety and toxicity and 1 month after operation of the 25th enroled patient, an interim safety analysis was performed by an IDMC.

### Statistical analysis

The primary endpoint was progression-free survival (PFS) at 1 year, defined as the absence of disease progression/relapse or death at 1 year ( ± 1 month) after registration.

Secondary endpoints included: treatment-related death during cetuximab-CT, cetuximab-RT and peri-operatively (until 30 days after surgery); tumour response after cetuximab-CT and after cetuximab-RT; complete pathological response (pCR); OS, defined as the time from registration until death due to any reason; adverse events (AEs); operability (based on a multidisciplinary tumour-board decision, under exhaustive evaluation of cardiac and pulmonary function according to ESTS/ATS guidelines); resection margins; failure pattern (defined as location of first progression).

Sample size was calculated based on PFS at 1 year. The PFS rate of ≤50% was considered uninteresting and ≥65% promising. Fleming-A’ Hern single-stage phase 2 procedure with a power of 80%, a one-sided significance level of 5% and a sample size of 69 evaluable patients was chosen.

For the primary endpoint, the PFS rate at 1 year together with its two-sided 90% CI was presented using the binary variable showing the information of progression at 1 year. PFS rate at 1 year was also calculated using the Kaplan–Meier estimator at 1 ear from registration together with both 90 and 95% two-sided CI, to be able to make comparisons with the results of the binary variable and to put our results in perspective to results in the published literature. Analyses were done with SAS version 9.4 and R version 3. The analysis of the primary endpoint was performed in both the intention-to-treat (ITT) population and the per protocol population (PP), defined as a subset of patients of the ITT population excluding patients who did not receive full trial treatment or patients who had major protocol violations in a prospectively planned exploratory analysis. For the secondary endpoints expressed as rates, the point estimates of the rate together with the associated two-sided 95% CI were calculated. For the secondary endpoints expressed as time-to-event endpoints, the median value was estimated using the Kaplan–Meier method along with a two-sided 95% CI. The type and number of events for each endpoint were presented descriptively by frequency and percentage. All data were collected and analysed at the SAKK Coordinating Centre in Berne, Switzerland. This trial is registered with ClinicalTrials.gov, number NCT01059188.

## Results

Between June 2010 and January 2016, 69 patients with resectable Stage IIIB NSCLC were included in the trial at 11 centres in Switzerland. One of the 69 patient was found to be misdiagnosed with lung cancer after having received full treatment and a supporting statistical analysis has been performed excluding this patient. Analysis for primary endpoint (1-year PFS) and toxicity is reported for the ITT population. However, 16 out of the accrued 69 patients were not included in the PP set (due to toxicity, early progression or death) and are not included in the analysis of secondary endpoints. Patients’ baseline and tumours characteristics are shown in Table 1. Twenty-seven (39%) patients had a T1–3N3M0 disease, 37 (54%) a T4N0–2M0 stage and 5 (7%) a T4N3M0 disease. The majority of patients had good performance status. Adenocarcinoma was the predominant histology (49%).

**Table 1:** Patient characteristics

Variable	Overall (*N* = 69)	
	*n*	(%)
Age (years)—median (min–max)	69	(36–73)
Sex		
Female	16	(23.2%)
Male	53	(76.8%)
Tumour IIIb stage		
T1 N3 M0	5	(7.2%)
T2 N3 M0	18	(26.1%)
T3 N3 M0	4	(5.8%)
T4 N0 M0	18	(26.1%)
T4 N1 M0	2	(2.9%)
T4 N2 M0	17	(24.6%)
T4 N3 M0	5	(7.2%)
Lymph-node staging by mediastinoscopy		
No	1	(1.4%)
Yes	57	(82.6%)
Not available	11	(15.9%)
Patient considered operable		
Yes	69	(100.0%)
WHO PS at entry		
0	46	(66.7%)
1	23	(33.3%)
Intervention planned at Tumour-board before inclusion		
Bilobectomy	11	(15.9%)
Lobectomy	36	(52.2%)
Pneumonectomy	15	(21.7%)
Missing	7	(10.1%)
Histology		
Adeno-NSCLC	34	(49.3%)
Large-cell NSCLC	1	(1.5%)
Poorly differentiated NSCLC	5	(7.2%)
Squamous NSCLC	28	(40.6%)
Missing^a^	1	(1.5%)
Pack-years of smoking—median (min–max)	40	(3–150)

^a^Histology of tumour could not be defined in pathology report as material was not sufficient for evaluation

**Fig. 2:**
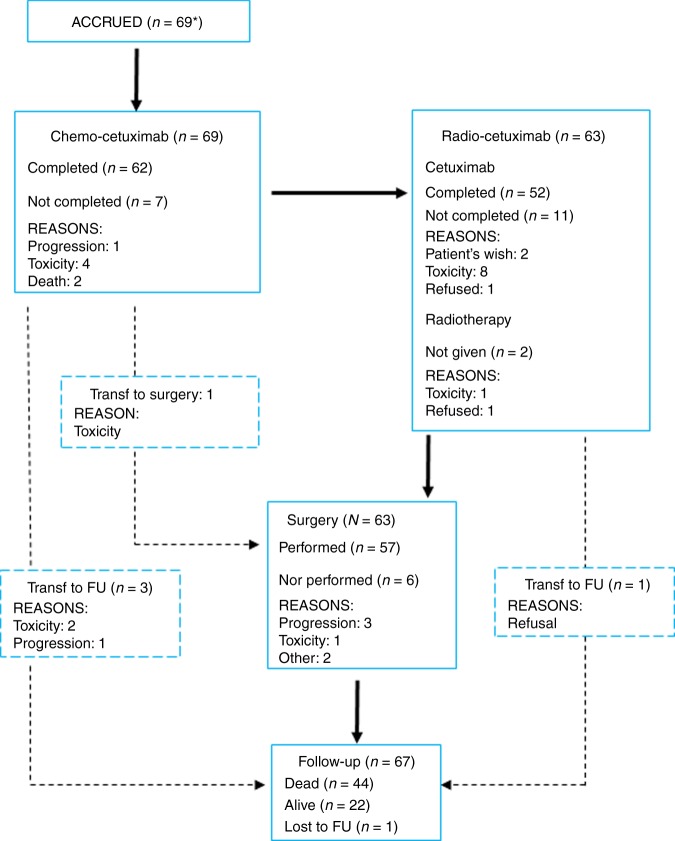
Flow Chart of the trial. Asterisk indicates a patient was mistakenly registered and was not included in the total accrual

Treatment delivery is presented in Fig. 2. Sixty-two out of 69 patients (90%) completed the three cycles of cetuximab-CT. A total of 197 cycles of cetuximab-CT were delivered with a median treatment duration of 63 days (21–83). Ninety percent of patients completed the three cycles of chemotherapy-cetuximab with a relative dose intensity for cetuximab of 90%, for cisplatin of 99.2%, for docetaxel of 98.5% (Supplementary Table 1). Eleven patients switched from cisplatin to carboplatin due to worsening of renal function (in four cases), hearing loss (in three cases), electrolytes disorders (three cases) and gastrointestinal toxicity (1 case). Reasons for not completing the three cycles were: death due to infection (in two cases), infection, haemoptysis, hepatitis B reactivation, oesophageal fungal infection and PD. Cetuximab-RT was planned for 63 patients. Two patients did not receive RT, due to toxicity and refusal after cetuximab-CT, respectively. RT schedule was administered for a median period of 20 days (19–25 days). Ninety-five percent of the 61 patients received radiotherapy per protocol with 22 fractions of 2 Gy with planed target volumes (respectively, with 30 Gy and 14 Gy). Cetuximab was given with a median duration of 21 days (7–42 days). In total, 52 (83%) completed the 3-weeks of cetuximab-radiotherapy. The response rate after cetuximab-CRT in these 52 patients was 64% (95% CI: 51–75%), see Table 2.

**Table 2:** Response after chemotherapy-cetuximab (CTC) and radiotherapy-cetuximab (RTC)

	Response	*N*	%	Response rate	95% CI
After CTC	PR	39	56.5	56.5%	(44.0–68.4%)
	SD	27	39.1		
	PD	1	1.4		
	NE	2^a^	2.9		
After RTC	PR	44	63.8	63.8%	(51.3–75.0%)
	SD	12	17.4		
	PD	3	4.3		
	NE	10	14.5		

^a^One patient stopped due to haemorrhagic tumour and one patient stopped due to death. These events are usually counted as *PD* (progressive disease), *PR* (partial response), *SD* (stable disease), *NE* (not evaluable)

Toxicity to cetuximab-CT was in the expected profile and range, including grade 3/4 neutropenia in 33% of patients, febrile neutropenia in 1%, renal function impairment grade 3 in 7%; grade 3 and 4 rash in 16% of patients, grade 3 diarrhoea in 15%. Drug related AEs leading to discontinuation of cetuximab-CT were observed in 9% of the patients. One patient died due to cerebral nocardiosis. Toxicity during cetuximab-RT included grade 2 esophagitis in 3% of patients, pneumonitis in 1.6% of patients. Thirteen percent of patients (8 out of 61 undergoing cetuximab-RT) discontinued the treatment due to adverse events (Supplementary Table 2).

The median time from registration to surgery was 17 weeks (range 8–21 weeks). Sixty-three out of the 69 ITT patients were considered operable (91%, 95% CI: 82–97%), but only 57 out of these 63 underwent surgery 90% (95% CI: 80–96%), related to disease progression (3), worsening of lung function (1) and absence of resectability (2). The median duration of patient hospitalisation for surgery was 13 days (3–113 days). The type of surgery (including bilobectomy, lobectomy or pneumonectomy), together with surgical outcomes, are summarised in Table 3 and Supplementary Table 3. Rate of pathologic complete response was 29% (95% CI: 19–41%) for all 69 patients. Calculated only for the R0/R1 resected patients, it was 35% (20 out of 57) (95% CI: 23–50%). No correlation was found between the rate of R0 resection and clinical nodal stage (N0–2 vs. N3; Fisher’s exact test *p* = 0.471).

**Table 3:** Surgery results

Variable	Overall (*N* = 69)	
	*n*	(%)
Operability		
No	6	8.7
Yes	57	82.6
NE	6	8.7
Resection		
R0	42	60.9
R1	14	20.3
NE	13	18.8
Pathologic complete response		
No	34	49.3
Yes	20	29.0
No R0/R1 resected	13	18.8
Missing	2	2.9

**Fig. 3:**
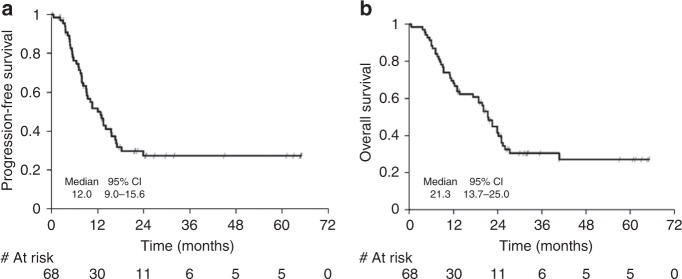
**a** Kaplan–Meier plot of progression-free survival (PFS). One-year PFS rate is 50% (95% CI: 37–62%; 90%CI: 39–60%). **b** Kaplan–Meier plot of overall survival (OS). Median OS was 21 months (95% CI: 14–25 months); estimated OS at 1, 2 and 3 years was 70% (57–79%), 41% (29–53%) and 30% (19–42%), respectively

Three patients died after surgery, two of them within 30 days after surgery with hypoxaemia and sepsis, and one 38 days after surgery with massive pulmonary haemorrhage. Thirty-day-postoperative mortality rate was 3.5% (2 out of 57). At the time of this analysis patients were followed-up for a median time of 32 months (IQR: 27–61 months). Median PFS was 12 months (95% CI: 9–16 months) (Fig. 3a). At the time of the analysis there were 43 events, mainly local or distant progression (86%). Among the 26 censored patients, five (19%) were followed for at least 5 years, ten (39%) were censored due to start of a new treatment and ten (39%) were still under follow-up.

One-year PFS rate, calculated according to the protocol as a binomial variable, was 38% (90% CI: 28–48%). However, using the Kaplan–Meier estimators, 1-year PFS rate was 50% (95% CI: 37–62%; 90%CI: 39–60%).

Median OS was 21 months (95% CI: 14–25 months). The Kaplan–Meier curve of OS can be found in Fig. 3b; estimated OS at 1, 2 and 3 years was 70% (57–79%), 41% (29–53%) and 30% (19–42%), respectively.

At the time of the analysis there were 46 deaths, mostly related to progression (80%). Among the 23 censored patients, 6 (26%) were followed for at least 5 years and 16 (70%) were still under follow-up.

## Discussion

About a third of patients with newly diagnosed NSCLC present with locally advanced disease. Optimal management of stage III NSCLC remains a matter of debate after several trials evaluating the role of surgery after C or CRT as compared to definitive radical CRT.^19,20,4,21–25^ The SAKK group previously randomised 232 stage IIIA/N2 NSCLC patients to induction chemo-radiotherapy vs. chemotherapy with a similar median event-free survival in the two groups.^25^ While radiotherapy did not add any benefit to induction chemotherapy followed by surgery, this trial suggested that one optimal local treatment is sufficient to treat resectable stage IIIA/N2 NSCLC.

The European Society of Medical Oncology (ESMO) does not recognise a clear benefit for one of the local treatments and the choice of local treatment modality may vary across countries and centres. For the American Society of Clinical Oncology (ASCO), definitive CRT is recommended in patients with good performance status with a median survival that typically reaches 15 to 20 months.

Multimodal approach of stage IIIB patients, including CRT and surgery reported a median OS of 17 months after CRT with surgery from the SWOG 8805 trial, with a survival rate of 39% at 2-years.^26^ Next the SAKK 16/01 trial, showed a median OS of 29 months and a 2-years OS of 52%. Such results led to the development of the here reported study (SAKK 16/08), resulting in a median OS of 21 months with a 2-year OS of 41%, reproducing the results of the SWOG trial. While in the SWOG trial, on the contrary of the SAKK 16/01 and SAKK 16/08 trials, supraclavicular lymph-node involvement and involvement of mediastinal structures were permitted, only 62% underwent surgery, compared to 71% from the SAKK 16/01 and 91% from the SAKK 16/08. This reflects the complexity of patients’ selection for surgery in stage IIIB. A higher rate of resectability might also be explained by the use of systematic PET-CT scans at staging in the SAKK 16/08.^27^ While PET was only used in the last 60% of patients enroled in the SAKK 16/01, SAKK 16/01 and 16/08 differed in the addition of cetuximab to induction treatment. Interestingly, the higher pathological complete response rate in the present trial compared to the SAKK 16/01 (29% vs. 13%, respectively), did neither result in an improved response rate to induction (64% vs. 59%), nor in 1-year PFS (50% vs. 54%) nor median OS (21 vs. 29 months). Toxicity to induction therapy was in the expected range and comparable to the SAKK 16/01 trial. Perioperative mortality in this trial was low and comparable to the SAKK 16/01 trial (4% in SAKK 16/08 vs. 6% in SAKK 16/01) and comparable to the SWOG 8805 trial (5.2%) The outcome of highly selected patients in both SAKK trials remains better than previously reported using definitive CRT.

The use of EGFR monoclonal antibodies in combination with frontline platinum-based chemotherapy allows for an improvement in OS, such benefit was sufficient for FDA to approve necitumumab in this setting, however, considered as a marginal benefit.^7,28^ Following encouraging preclinical and early clinical trials results, controlled randomised trials were unable to demonstrate improved outcome of the cetuximab arms combined with CRT compared to CRT alone in stage III NSCLC.^29,30^ The treatment regimen presented here (SAKK 16/08) however differs from the above-mentioned studies due to the inclusion restricted to selected resectable stage IIIB patients, the surgical approach and the absence of maintenance strategy, in order to limit perioperative toxicity. As EGFR expression in tumours might vary after induction,^31,32^ maintenance cetuximab might influence the outcome of patients with high EGFR expression.^30^ In the present study, we did not assess EGFR expression status of patients due to potential bias related to the small size of the cohort.

Compared to historical series of stage IIIB treated with CRT therapy, our study shows a high disease control rate. However, there are some limitations due to the absence of randomisation with a control group. Moreover, patients were highly selected, being candidates for surgery with good performance status, as well as pulmonary reserve and heart function. Our results suggest that careful patient selection and intensive multimodal therapy can lead to better results than the ones obtained with definitive CRT, and that stage IIIB disease can be cured.

Novel approaches in non-resectable stage III NSCLCs have been recently reported in the PACIFIC trial where patients were treated by CRT and randomised to receive a PD-L1 inhibitor, namely durvalumab.^33,34^ The primary endpoint of progression-free survival was met with a median of 17.2 months vs. 5.6 months in the durvalumab group compared to control and a 2 year OS of 63%. While these patients were considered non-resectable based on local standards, our study offers novel perspectives in the multimodal management of stage III NSCLC and might impact the debate about the role of surgery in stage III NSCLC. Next generation of trials combining immunotherapies in the context of stage III definitive CRT are in preparation or recruitment stage. Novel treatment protocols that include induction chemo-immunotherapy followed by surgery and immunotherapy maintenance administration are also under investigation in early NSCLC, including stage IIIA. Knowing the high risk of distant relapse in stage III NSCLC, combination of an optimal local control and immunotherapy-mediated immunogenic cell death will certainly provide the most interesting long-term benefit in locally advanced lung cancer.

## Supplementary information

Supplementary tables

## Data Availability

Data supporting the results reported in the article can be found including, where applicable, hyperlinks to publicly archived datasets analysed or generated during the study.

## References

[1] Goldstraw P, Chansky K, Crowley J, Rami-Porta R, Asamura H, Eberhardt WE (2016). The IASLC Lung Cancer Staging Project: Proposals for revision of the TNM stage groupings in the forthcoming (Eighth) edition of the TNM classification for lung cancer. J. Thorac. Oncol..

[2] Peters S, Weder W, Dafni U, Kerr KM, Bubendorf L, Meldgaard P (2014). Lungscape: resected non-small-cell lung cancer outcome by clinical and pathological parameters. J. Thorac. Oncol..

[3] Albain KS, Rusch VW, Crowley JJ, Rice TW, Turrisi AT, Weick JK (1995). Concurrent cisplatin/etoposide plus chest radiotherapy followed by surgery for stages IIIA (N2) and IIIB non-small-cell lung cancer: mature results of Southwest Oncology Group phase II study 8805. J. Clin. Oncol..

[4] Stupp R, Mayer M, Kann R, Weder W, Zouhair A, Betticher DC (2009). Neoadjuvant chemotherapy and radiotherapy followed by surgery in selected patients with stage IIIB non-small-cell lung cancer: a multicentre phase II trial. Lancet Oncol..

[5] Grunenwald DH, Andre F, Le Pechoux C, Girard P, Lamer C, Laplanche A (2001). Benefit of surgery after chemoradiotherapy in stage IIIB (T4 and/or N3) non-small cell lung cancer. J. Thorac. Cardiovasc. Surg..

[6] Ichinose Y, Fukuyama Y, Asoh H, Ushijima C, Okamoto T, Ikeda J (2003). Induction chemoradiotherapy and surgical resection for selected stage IIIB non-small-cell lung cancer. Ann. Thorac. Surg..

[7] Pirker R, Pereira JR, Szczesna A, von Pawel J, Krzakowski M, Ramlau R (2009). Cetuximab plus chemotherapy in patients with advanced non-small-cell lung cancer (FLEX): an open-label randomised phase III trial. Lancet.

[8] Schmidt-Ullrich RK, Valerie KC, Chan W, McWilliams D (1994). Altered expression of epidermal growth factor receptor and estrogen receptor in MCF-7 cells after single and repeated radiation exposures. Int. J. Radiat. Oncol. Biol. Phys..

[9] Huang SM, Bock JM, Harari PM (1999). Epidermal growth factor receptor blockade with C225 modulates proliferation, apoptosis, and radiosensitivity in squamous cell carcinomas of the head and neck. Cancer Res..

[10] Lammering G, Hewit TH, Hawkins WT, Contessa JN, Reardon DB, Lin PS (2001). Epidermal growth factor receptor as a genetic therapy target for carcinoma cell radiosensitization. J. Natl. Cancer. Inst..

[11] Blumenschein GR, Paulus R, Curran WJ, Robert F, Fossella F, Werner-Wasik M (2011). Phase II study of cetuximab in combination with chemoradiation in patients with stage IIIA/B non-small-cell lung cancer: RTOG 0324. J. Clin. Oncol..

[12] Hallqvist A, Wagenius G, Rylander H, Brodin O, Holmberg E, Loden B (2011). Concurrent cetuximab and radiotherapy after docetaxel-cisplatin induction chemotherapy in stage III NSCLC: satellite--a phase II study from the Swedish Lung Cancer Study Group. Lung Cancer.

[13] De Leyn P, Lardinois D, Van Schil PE, Rami-Porta R, Passlick B, Zielinski M (2007). ESTS guidelines for preoperative lymph node staging for non-small cell lung cancer. Eur. J. Cardiothorac. Surg..

[14] Martini N (1995). Mediastinal lymph node dissection for lung cancer. The Memorial experience. Chest. Surg. Clin. N. Am..

[15] Brunelli A, Charloux A, Bolliger CT, Rocco G, Sculier JP, Varela G (2009). The European Respiratory Society and European Society of Thoracic Surgeons clinical guidelines for evaluating fitness for radical treatment (surgery and chemoradiotherapy) in patients with lung cancer. Eur. J. Cardiothorac. Surg..

[16] Brunelli A, Charloux A, Bolliger CT, Rocco G, Sculier JP, Varela G (2009). ERS/ESTS clinical guidelines on fitness for radical therapy in lung cancer patients (surgery and chemo-radiotherapy). Eur. Respir. J..

[17] Colice GL, Shafazand S, Griffin JP, Keenan R, Bolliger CT (2007). American College of Chest P. Physiologic evaluation of the patient with lung cancer being considered for resectional surgery: ACCP evidenced-based clinical practice guidelines (2nd edition). Chest.

[18] Fleisher LA, Beckman JA, Brown KA, Calkins H, Chaikof E, Fleischmann KE (2007). ACC/AHA 2007 guidelines on perioperative cardiovascular evaluation and care for noncardiac surgery: a report of the American College of Cardiology/American Heart Association Task Force on Practice Guidelines (Writing Committee to Revise the 2002 Guidelines on Perioperative Cardiovascular Evaluation for Noncardiac Surgery): developed in collaboration with the American Society of Echocardiography, American Society of Nuclear Cardiology, Heart Rhythm Society, Society of Cardiovascular Anesthesiologists, Society for Cardiovascular Angiography and Interventions, Society for Vascular Medicine and Biology, and Society for Vascular Surgery. Circulation.

[19] Albain KS, Swann RS, Rusch VW, Turrisi AT, Shepherd FA, Smith C (2009). Radiotherapy plus chemotherapy with or without surgical resection for stage III non-small-cell lung cancer: a phase III randomised controlled trial. Lancet.

[20] Eberhardt WE, Pottgen C, Gauler TC, Friedel G, Veit S, Heinrich V (2015). Phase III study of surgery versus definitive concurrent chemoradiotherapy boost in patients with resectable stage IIIA(N2) and selected IIIB non-small-cell lung cancer after induction chemotherapy and concurrent chemoradiotherapy (ESPATUE). J. Clin. Oncol..

[21] Cerfolio RJ, Bryant AS, Spencer SA, Bartolucci AA (2005). Pulmonary resection after high-dose and low-dose chest irradiation. Ann. Thorac. Surg..

[22] Uy KL, Darling G, Xu W, Yi QL, De Perrot M, Pierre AF (2007). Improved results of induction chemoradiation before surgical intervention for selected patients with stage IIIA-N2 non-small cell lung cancer. J. Thorac. Cardiovasc. Surg..

[23] Betticher DC, Hsu Schmitz SF, Totsch M, Hansen E, Joss C, von Briel C (2003). Mediastinal lymph node clearance after docetaxel-cisplatin neoadjuvant chemotherapy is prognostic of survival in patients with stage IIIA pN2 non-small-cell lung cancer: a multicenter phase II trial. J. Clin. Oncol..

[24] Weder W, Collaud S, Eberhardt WE, Hillinger S, Welter S, Stahel R (2010). Pneumonectomy is a valuable treatment option after neoadjuvant therapy for stage III non-small-cell lung cancer. J. Thorac. Cardiovasc. Surg..

[25] Pless M, Stupp R, Ris HB, Stahel RA, Weder W, Thierstein S (2015). Induction chemoradiation in stage IIIA/N2 non-small-cell lung cancer: a phase 3 randomised trial. Lancet.

[26] Rusch VW, Albain KS, Crowley JJ, Rice TW, Lonchyna V, Mckenna R (1994). Neoadjuvant therapy-a novel and effective treatment for Stage IIIB nonsmall cell lung-cancer. Ann. Thoracic Surg..

[27] Fischer B, Lassen U, Mortensen J, Larsen S, Loft A, Bertelsen A (2009). Preoperative staging of lung cancer with combined PET-CT. N. Engl. J. Med..

[28] Thatcher N, Hirsch FR, Luft AV, Szczesna A, Ciuleanu TE, Dediu M (2015). Necitumumab plus gemcitabine and cisplatin versus gemcitabine and cisplatin alone as first-line therapy in patients with stage IV squamous non-small-cell lung cancer (SQUIRE): an open-label, randomised, controlled phase 3 trial. Lancet Oncol..

[29] Govindan R, Bogart J, Stinchcombe T, Wang X, Hodgson L, Kratzke R (2011). Randomized phase II study of pemetrexed, carboplatin, and thoracic radiation with or without cetuximab in patients with locally advanced unresectable non-small-cell lung cancer: Cancer and Leukemia Group B trial 30407. J. Clin. Oncol..

[30] Bradley JD, Paulus R, Komaki R, Masters G, Blumenschein G, Schild S (2015). Standard-dose versus high-dose conformal radiotherapy with concurrent and consolidation carboplatin plus paclitaxel with or without cetuximab for patients with stage IIIA or IIIB non-small-cell lung cancer (RTOG 0617): a randomised, two-by-two factorial phase 3 study. Lancet Oncol..

[31] Withers HR, Taylor JM, Maciejewski B (1988). The hazard of accelerated tumor clonogen repopulation during radiotherapy. Acta Oncol..

[32] Eicheler W, Krause M, Hessel F, Zips D, Baumann M (2005). Kinetics of EGFR expression during fractionated irradiation varies between different human squamous cell carcinoma lines in nude mice. Radiother. Oncol..

[33] Antonia SJ, Villegas A, Daniel D, Vicente D, Murakami S, Hui R (2017). Durvalumab after chemoradiotherapy in stage III non-small-cell lung cancer. N. Engl. J. Med..

[34] Antonia SJ, Villegas A, Daniel D, Vicente D, Murakami S, Hui R (2018). Overall survival with durvalumab after chemoradiotherapy in stage III NSCLC. N. Engl. J. Med..

