# Supplementary material for: Preoperative chemotherapy and radiotherapy concomitant to cetuximab in resectable stage IIIB NSCLC: a multicentre phase 2 trial (SAKK 16/08)
Source: Br J Cancer. 2019 Apr 16;120(10):968–74. doi: 10.1038/s41416-019-0447-0 (PMC6734655; doi:10.1038/s41416-019-0447-0)
Supplement: Supplementary file 1 — Supplementary tables [file 41416_2019_447_MOESM1_ESM.docx]

| **Supplementary Table 1. Relative total dose intensity (RTDI) of chemotherapy-cetuximab**   \|  \| \| \| \| \| \| \| --- \| --- \| --- \| --- \| --- \| --- \| \|  \| \| RTDI (%) \| \| \| \| \| Treatment \| N \| Mean \| Median \| Min \| Max \| \| Cetuximab \| 69 \| 84.1 \| 90.0 \| 13.8 \| 108 \| \| Cisplatin \| 67 \| 97.7 \| 99.2 \| 81.0 \| 105 \| \| Docetaxel \| 69 \| 93.6 \| 98.5 \| 55.3 \| 105 \|   **Supplementary Table 2. Side effects to chemotherapy-cetuximab**   \|  \| **Overall (N=69)** \| \| \| \| \| \| \| \| \| \| \| --- \| --- \| --- \| --- \| --- \| --- \| --- \| --- \| --- \| --- \| --- \| \|  \| **Grade** \| \| \| \| \| \| \| \| \| \| \|  \| **1** \| \| **2** \| \| **3** \| \| **4** \| \| **5** \| \| \| **Category** \| **n** \| **%** \| **n** \| **%** \| **n** \| **%** \| **n** \| **%** \| **n** \| **%** \| \| Allergy/Immunology \| - \| - \| 4 \| 5.8 \| 2 \| 2.9 \| 1 \| 1.4 \| - \| - \| \| Auditory/Ear \| 1 \| 1.4 \| 8 \| 11.6 \| 1 \| 1.4 \| - \| - \| - \| - \| \| Blood/Bone marrow \| 25 \| 36.2 \| 3 \| 4.3 \| 17 \| 24.6 \| 9 \| 13.0 \| - \| - \| \| Cardiac Arrhythmia \| 4 \| 5.8 \| 1 \| 1.4 \| 2 \| 2.9 \| - \| - \| - \| - \| \| Cardiac General \| - \| - \| 4 \| 5.8 \| 1 \| 1.4 \| 1 \| 1.4 \| - \| - \| \| Coagulation \| - \| - \| - \| - \| 1 \| 1.4 \| - \| - \| - \| - \| \| Constitutional symptoms \| 9 \| 13.0 \| 36 \| 52.2 \| 13 \| 18.8 \| 2 \| 2.9 \| - \| - \| \| Dermatology/Skin \| 18 \| 26.1 \| 29 \| 42.0 \| 13 \| 18.8 \| 1 \| 1.4 \| - \| - \| \| Gastrointestinal \| 13 \| 18.8 \| 28 \| 40.6 \| 24 \| 34.8 \| - \| - \| - \| - \| \| Hemorrhage/Bleeding \| 9 \| 13.0 \| 2 \| 2.9 \| 1 \| 1.4 \| - \| - \| - \| - \| \| Hepatobiliary/Pancreas \| - \| - \| 1 \| 1.4 \| 1 \| 1.4 \| - \| - \| - \| - \| \| Infection \| 1 \| 1.4 \| 8 \| 11.6 \| 10 \| 14.5 \| 3 \| 4.3 \| - \| - \| \| Lymphatics \| 8 \| 11.6 \| 5 \| 7.2 \| - \| - \| - \| - \| - \| - \| \| Metabolic/Laboratory \| 12 \| 17.4 \| 7 \| 10.1 \| 8 \| 11.6 \| 5 \| 7.2 \| - \| - \| \| Musculoskeletal/Soft Tissue \| - \| - \| 1 \| 1.4 \| - \| - \| - \| - \| - \| - \| \| Neurology \| 13 \| 18.8 \| 10 \| 14.5 \| 3 \| 4.3 \| - \| - \| - \| - \| \| Ocular/Visual \| 1 \| 1.4 \| 1 \| 1.4 \| - \| - \| - \| - \| - \| - \| \| Pain \| 6 \| 8.7 \| 16 \| 23.2 \| 4 \| 5.8 \| 3 \| 4.3 \| - \| - \| \| Pulmonary/Upper Respiratory \| 16 \| 23.2 \| 13 \| 18.8 \| 6 \| 8.7 \| 1 \| 1.4 \| - \| - \| \| Renal/Genitourinary \| 2 \| 2.9 \| 1 \| 1.4 \| 5 \| 7.2 \| - \| - \| - \| - \| \| Vascular \| - \| - \| 1 \| 1.4 \| 3 \| 4.3 \| - \| - \| - \| - \|   Supplementary Table 3. Additional information about surgery | | |
| --- | --- | --- | --- | --- | --- | --- | --- | --- | --- | --- | --- | --- | --- | --- | --- | --- | --- | --- | --- | --- | --- | --- | --- | --- | --- | --- | --- | --- | --- | --- | --- | --- | --- | --- | --- | --- | --- | --- | --- | --- | --- | --- | --- | --- | --- | --- | --- | --- | --- | --- | --- | --- | --- | --- | --- | --- | --- | --- | --- | --- | --- | --- | --- | --- | --- | --- | --- | --- | --- | --- | --- | --- | --- | --- | --- | --- | --- | --- | --- | --- | --- | --- | --- | --- | --- | --- | --- | --- | --- | --- | --- | --- | --- | --- | --- | --- | --- | --- | --- | --- | --- | --- | --- | --- | --- | --- | --- | --- | --- | --- | --- | --- | --- | --- | --- | --- | --- | --- | --- | --- | --- | --- | --- | --- | --- | --- | --- | --- | --- | --- | --- | --- | --- | --- | --- | --- | --- | --- | --- | --- | --- | --- | --- | --- | --- | --- | --- | --- | --- | --- | --- | --- | --- | --- | --- | --- | --- | --- | --- | --- | --- | --- | --- | --- | --- | --- | --- | --- | --- | --- | --- | --- | --- | --- | --- | --- | --- | --- | --- | --- | --- | --- | --- | --- | --- | --- | --- | --- | --- | --- | --- | --- | --- | --- | --- | --- | --- | --- | --- | --- | --- | --- | --- | --- | --- | --- | --- | --- | --- | --- | --- | --- | --- | --- | --- | --- | --- | --- | --- | --- | --- | --- | --- | --- | --- | --- | --- | --- | --- | --- | --- | --- | --- | --- | --- | --- | --- | --- | --- | --- | --- | --- | --- | --- | --- | --- | --- | --- | --- | --- | --- | --- | --- | --- | --- | --- | --- | --- | --- | --- | --- | --- | --- | --- | --- | --- | --- | --- | --- | --- | --- | --- | --- | --- | --- | --- | --- | --- | --- | --- | --- | --- | --- | --- | --- | --- | --- | --- | --- | --- | --- | --- | --- | --- | --- | --- | --- | --- | --- | --- | --- | --- | --- | --- | --- | --- | --- | --- | --- | --- | --- | --- | --- |
|  | **Overall (N=57)** | |
|  | **n** | **(%)** |
| Pneumonectomy left |  |  |
| . No | 43 | (75.4%) |
| . Yes | 14 | (24.6%) |
| Pneumonectomy right |  |  |
| . No | 53 | (93.0%) |
| . Yes | 4 | (7.0%) |
| Lobectomy |  |  |
| . No | 29 | (50.9%) |
| . Yes | 28 | (49.1%) |
| Bi-lobectomy |  |  |
| . No | 47 | (82.5%) |
| . Yes | 10 | (17.5%) |
| Chest wall |  |  |
| . No | 50 | (87.7%) |
| . Yes | 7 | (12.3%) |
| Carina (partial/complete) |  |  |
| . No | 54 | (94.7%) |
| . Yes | 3 | (5.3%) |
| Pericardium |  |  |
| . No | 49 | (86.0%) |
| . Yes | 8 | (14.0%) |
| Vertebral body |  |  |
| . No | 54 | (94.7%) |
| . Yes | 3 | (5.3%) |
| Great vessels |  |  |
| . No | 48 | (84.2%) |
| . Yes | 9 | (15.8%) |
| Other |  |  |
| . No | 49 | (86.0%) |
| . Missing | 1 | (1.8 %) |
| . Yes | 7 | (12.3%) |
| *Chondrotomy of the 4. rib* |  |  |
| *Ribs 1,2,3,4 / Pleura parietalis* |  |  |
| *Mediastinum invasion* |  |  |
| *Wedgeresection apical lower lobe* |  |  |
| *Oesophageal wall resection R0*  *Upper lobe sleeve resection* |  |  |
| *Thymus* |  |  |
| Right upper & lower paratracheal |  |  |
| . No | 13 | (22.8%) |
| . Yes | 44 | (77.2%) |
| Aortopulmonal |  |  |
| . No | 30 | (52.6%) |
| . Yes | 27 | (47.4%) |
| Anterior mediastinal |  |  |
| . No | 32 | (56.1%) |
| . Yes | 25 | (43.9%) |
| Subcarinal |  |  |
| . No | 9 | (15.8%) |
| . Yes | 48 | (84.2%) |
| Paraoesophageal |  |  |
| . No | 20 | (35.1%) |
| . Yes | 37 | (64.9%) |
| Right tracheobronchial |  |  |
| . No | 29 | (50.9%) |
| . Yes | 28 | (49.1%) |
| Left tracheobronchial |  |  |
| . No | 39 | (68.4%) |
| . Yes | 18 | (31.6%) |
| Left upper & lower paratracheal |  |  |
| . No | 18 | (31.6%) |
| . Yes | 19 | (33.3%) |
| . Missing | 20 | (35.1%) |
| Prevascular & retrotracheal |  |  |
| . No | 28 | (49.1%) |
| . Yes | 8 | (14.0%) |
| Missing | 21 | (36.8%) |
